# Portable System for Box Volume Measurement Based on Line-Structured Light Vision and Deep Learning

**DOI:** 10.3390/s19183921

**Published:** 2019-09-11

**Authors:** Tao Peng, Zhijiang Zhang, Yingjie Song, Fansheng Chen, Dan Zeng

**Affiliations:** 1 Key Laboratory of Specialty Fiber Optics and Optical Access Networks, Joint International Research Laboratory of Specialty Fiber Optics and Advanced Communication, Shanghai Institute for Advanced Communication and Data Science, Shanghai University, 99 Shangda Road, Shanghai 200444, China; 2 Key Laboratory of Intelligent Infrared Perception, Chinese Academy of Sciences, Shanghai 200444, China

**Keywords:** volume measurement, line-structured light, edge detection, deep learning

## Abstract

Portable box volume measurement has always been a popular issue in the intelligent logistic industry. This work presents a portable system for box volume measurement that is based on line-structured light vision and deep learning. This system consists of a novel 2 × 2 laser line grid projector, a sensor, and software modules, with which only two laser-modulated images of boxes are required for volume measurement. For laser-modulated images, a novel end-to-end deep learning model is proposed by using an improved holistically nested edge detection network to extract edges. Furthermore, an automatic one-step calibration method for the line-structured light projector is designed for fast calibration. The experimental results show that the measuring range of our proposed system is 100–1800 mm, with errors less than ±5.0 mm. Theoretical analysis indicates that within the measuring range of the system, the measurement uncertainty of the measuring device is ±0.52 mm to ±4.0 mm, which is consistent with the experimental results. The device size is 140 mm × 35 mm × 35 mm and the weight is 110 g, thus the system is suitable for portable automatic box volume measurement.

## 1. Introduction

Box volume measurement is important for many sectors, including logistics, transportation, and production, and it can assist in designing, packaging, and allocating strategies. Fast, intelligent, accurate, and automatic volume measurement can improve efficiency and reduce labor intensity. User-friendly and cost-effective systems are also vital for box volume measurement. 

As previously mentioned, a practical measurement system for box volume should have the following characteristics: (1) relatively small to be handled gracefully, (2) wide measuring range, (3) high measurement accuracy, (4) stable and robust, and (5) easy to use and flexible.

At present, the research hotspots of large-scale measurement methods with three-dimensional (3D) geometric dimension focus on non-contact 3D measurement methods based on computer vision technology. This method has a rigorous theoretical basis, a large range of elasticity, high measurement accuracy and efficiency, no rigid requirement for the spatial relationship between the measuring device and measured object, good robustness, and non-contact measurement. Thus, this method is a feasible solution for solving large-scale 3D geometric measurement.

With the development of computer vision technology, object volume can be calculated while using new technology and sensors [1,2,3]. Many advanced sensors, such as stereovision, time-of-flight (ToF) camera, and structured-light vision sensor, can represent spatial and color information from natural objects, thereby playing a crucial role in the development of industrial automation measurement.

A method for the dimension measurement and inspection of cuboidal objects (boxes) with a ToF camera was described in [4], with an average error of 5 mm. The same ToF camera was used in [5] to build a system for computing the volume of cuboidal objects with an accuracy of 8 mm. The ToF technology can obtain depth information in real time by calculating the time that it takes for a pulse of energy to travel from its transmitter to the object surface and then back to the receiver. The ToF camera technique, due to its robustness and popularity, has been widely studied and applied in industries [6,7]. The dimensional measurement methods for objects that are based on stereovision have also been widely used. A stereovision technique for accurately measuring the distance and size (height and width) of an object in view was introduced in [8]. Ge et al. [9] proposed a method of broccoli seedling recognition in natural environments based on binocular stereovision. As binocular cameras heavily rely on image feature matching, the effect is poor under dark or overexposed lighting. In addition, if the measured scene lacks texture, then extracting and matching the features are difficult. In addition, a binocular stereocamera uses complex correlation algorithm, which is time consuming. The depth calculation of ToF is unaffected by the grayscale and features of the object surface, and the ToF can accurately perform 3D detection. The depth calculation accuracy of ToF does not change with the change in distance. The measurement accuracy can reach the mm level by using an advanced ToF camera and algorithm, as previously mentioned [4,5].

**Figure 1:**
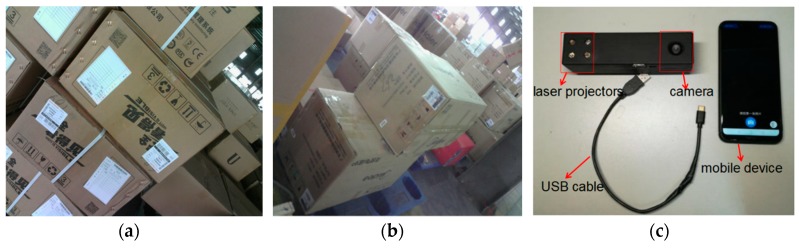
Box in the distribution center and design prototype; (**a**,**b**) show the box in the distribution, and (**c**) shows the proposed system prototype.

Recently, the technique of computer vision and structured light (SL) measurement has been widely applied in many fields of high-precision measurement, due to its simple structure. Triangulation-based visual sensors are popular for measurement in various industries. They have many advantages, such as non-contact, high-precision, rapid, and automated measurements [10,11,12,13]. Fernandes et al. [14] presented an approach that is based on projective geometry; they computed the box dimensions by using data that were extracted from the box silhouette and the projection of two parallel laser beams on one of the imaged faces of the box. Wang et al. [15] proposed a handheld 3D laser scanning system that consists of a binocular stereovision and line laser projector for measuring large-sized objects on site. Pan et al. [16] proposed a wheel size measurement framework that is based on a structured-light vision sensor, which has high precision and reliability and is suitable for highly reflective conditions. In the present study, we develop a novel volume measurement system for a box that contains high-resolution color digital cameras and line-structured lights and that works indoors and outdoors. Figure 1c shows the designed device for box volume measurement. The device size is 140 mm × 35 mm × 35 mm and the weight is 110 g, thereby easily meeting the requirements of stability and portability. The line-structured light projectors emit laser planes onto the box face, and the laser planes intersect with the face of the measured box and form laser stripes in the laser-modulated image. As the face of the measured box modulates the laser stripes, the image processing algorithm can calculate the dimension information of the box on the basis of the laser triangulation principle and some key points. Thus, our method calculates the volume of boxes from two laser-modulated images (two adjacent faces of the box), and the technique mainly includes two aspects: (1) calibration technology of the vision sensor and (2) the extraction of the box silhouette to obtain the key points from the laser-modulated images.

The measuring range and precision can be settled by studying the calibration algorithms [17,18,19,20,21,22,23,24,25]. Silhouette extraction is another key issue for laser-modulated image processing. Figure 1a,b show the differences in the appearances of boxes and backgrounds. Therefore, we need a robust algorithm to extract edges for laser-modulated images. The vigorous development of deep learning provides us with possible solutions. The deep fully convolutional neural network (FCNN) [26] has been proposed as a solution to similar problems. FCNN has reached the level of human beings in multi-level edge and target boundary detections in natural images [27,28], which leads to a breakthrough in semantic segmentation [29]. Song et al. [30] proposed an algorithm for detecting building corners in aerial images by training a DeepLab network; they achieved excellent results. Xie et al. [31] developed an efficient and accurate edge detector, namely, holistically nested edge detection (HED), which performs well in edge detection tasks. Inspired by HED, the present study trains an end-to-end deep learning model for the laser-modulated image by adopting an improved holistically nested edge detection (IHED) network. 

This work presents an approach for computing the box volume from only two laser-modulated images in a completely automatic manner. The method uses information that was extracted from the structure edges of the measured boxes, which can be computed when at least two of their faces are projected by the laser projector. We demonstrate this approach by developing a prototype visual sensor for calculating the box volume online (Figure 1c).

The main contributions of this study are as follows:

1. Visual sensor. A portable mechanical structure for box volume measurement is proposed with an accuracy of ±5.0 mm and measuring range of 10–1800 mm (Section 2 and Section 3.1).
2. Box volume measurement algorithm. A novel algorithm for calculating the box volume in a completely automatic manner online is presented (Section 3.2).
3. Calibration. A novel calibration method for the automatic calibration of our system is proposed. This method performs camera and laser projector calibrations in a single step, thus avoiding the digitalization of a reference sphere to obtain extrinsic parameters (Section 3.3).
4. Edge detection. A total of 40,000 labeled laser-modulated images are collected. With this box edge detection database, a novel end-to-end deep learning architecture that is based on IHED is proposed and it has achieved excellent performance (Section 3.4).

The paper is organized, as follows. Section 2 presents a brief overview and operating instructions of the visual sensor of the system. Our new approach for measuring the box volume is investigated in detail in Section 3. The experimental results and discussions are presented in Section 4. Finally, conclusions are drawn in Section 5. 

## 2. Overview

**Figure 2:**
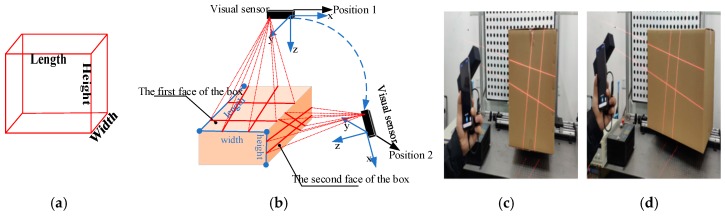
Schematic of the measurement system and two images captured by the system; (**a**) the box model; (**b**) the measurement method of the visual sensor and measured box; (**c**,**d**) the captured images for the measured box.

Figure 1c displays the proposed system. High-precision sensors and strict measurement rules achieve high-accuracy measurement. Figure 2b shows the measurement method of the visual sensor and measured box. The detailed workflow is listed, as follows:

1. Solving parameters: Before using the system, we obtain the parameters by using our calibration method (Section 3.3).
2. Data collection: The visual sensor connected to a portable mobile device is used. Two images of any two adjacent faces of the box are obtained. The four modulated laser stripes should intersect the four edges of the box face, as shown in Figure 2c,d.
3. Volume measurement: The system will automatically process the collected images and then obtain the box length, width, and height. Finally, the system automatically obtains the volume of the measured box.

**Figure 3:**
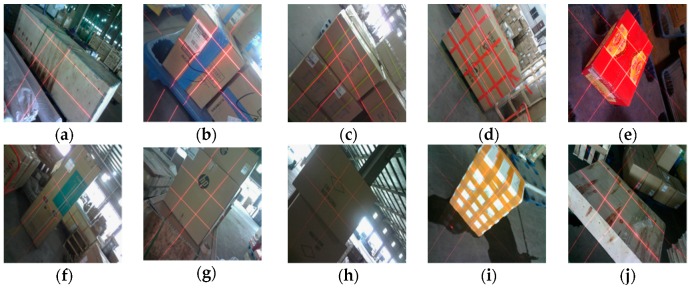
Images that are captured by our device in the distribution center; (**a**–**j**) show the different images captured by our system.

The regular logistics box volume is an important indicator of the freight that was collected in the logistics industry. The box length, width, and height should be measured to determine the box volume. Certain difficulties exist in volume measurement system, which are reflected in the following four aspects: (1) The environment inside the distribution center is complex and it suffers from different illumination information (Figure 3a,c,g,h). (2) Logistics boxes have varied sizes, and the box length ranges from 10 mm to 1800 mm (Figure 3a,b,e,f,j). (3) Laser-modulated images are influenced by variations in box materials, color, and appendages (Figure 3b,d,f,h,i). (4) Non-contact and portable measurements are required. 

To solve the abovementioned problems, we model the boxes as parallelepipeds, as shown in Figure 2a. The volume of a parallelepiped can be calculated while using the 3D coordinates of the vertices with two arbitrary adjacent faces of the box. The 3D coordinates of a box’s face can be obtained on the basis of the intersection of the laser lines and the edges of the box’s face. Thus, the edge of the laser line and box edges on the laser-modulated images must be extracted before we can calculate the volume of the measured box (Section 3.4), and then the equations of the laser planes of the laser projector and the camera parameters must be obtained (Section 3.3).

**Figure 4:**
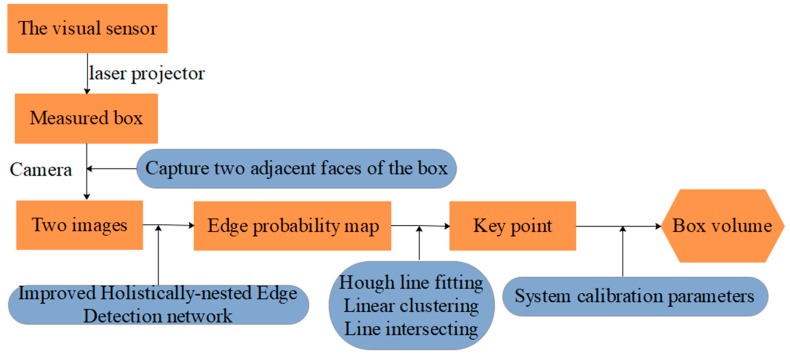
Workflow of the box volume measurement system.

Our portable system for box volume measurement that is based on line-structured light vision and deep learning only requires two laser-modulated box images for the measurement. Figure 4 depicts the scheme behind the proposed solution. Before the measurement, we obtain the parameters by using our calibration method and write the parameters to the device. We input the two laser-modulated images into the designed network to generate the edge probability map. Subsequently, we obtain the coordinates of key points of the box face through a simple image processing of the edge probability map. We can obtain the box volume combined with the calibration parameters and key points.

## 3. Mathematical Modeling

### 3.1. Design of the Visual Sensor Measurement System

**Figure 5:**
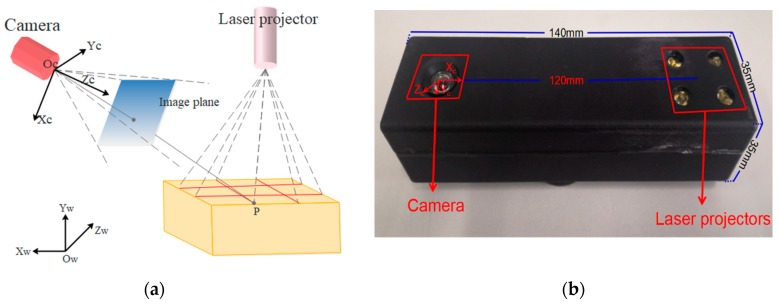
(**a**) Measurement schematics of the proposed volume measurement system; and, (**b**) volume measurement device that we designed.

The portable volume measurement system that was proposed in this work consists of a 2 × 2 laser line grid projector and high-resolution camera, as shown in Figure 5b; it has a low computational cost. Table 1 lists the detailed parameters of the visual sensor. The size of the designed device is 140 mm × 35 mm × 35 mm, and the weight is 110 g. The baseline length of the device is 120 mm, thereby easily meeting the requirements of stability and portability. Furthermore, connection to other mobile devices, such as a mobile phone or pad, is convenient.

Figure 5a presents the measurement schematics of the proposed volume measurement system. $O_{w}-X_{w}Y_{w}Z_{w}$ is the world coordinate system (WCS), and $O_{c}-X_{c}Y_{c}Z_{c}$ is the camera coordinate system (CCS). The laser stripes are projected onto the box face through a laser projector. The camera captures the laser stripes that are modulated by the box faces. Afterwards, the laser-modulated images are captured. However, the four modulated laser stripes must intersect the four edges of the box faces.

### 3.2. Geometric Model

**Figure 6:**
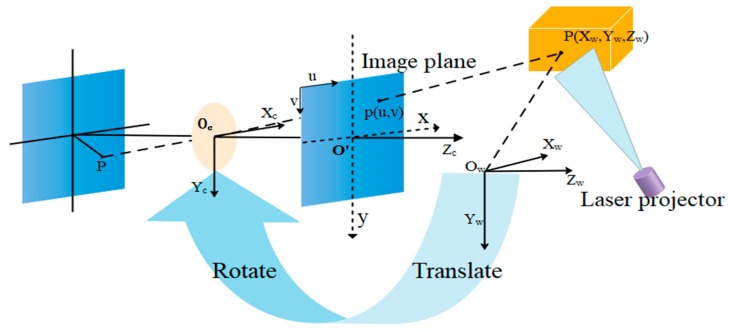
Perspective projection model of the visual sensor.

Camera mapping coordinate points in a 3D world to a two-dimensional (2D) image plane can be described while using a pinhole model [32]. Figure 6 shows the perspective projection relationship between 3D space point and 2D image point in the pinhole camera model.

The projection from a 3D point $P(x_{w},y_{w},z_{w})$ in the WCS to a 2D image point p(u,v) in the image plane is expressed by the following equation:

(1) $$\begin{array}{l} \rho \left[\begin{array}{c} u\\ \nu \\ 1 \end{array}\right]=\left[\begin{array}{cccc} \alpha & \delta & u_{0} & 0\\ 0 & \beta & \nu_{0} & 0\\ 0 & 0 & 1 & 0 \end{array}\right]\left[\begin{array}{cc} R & T\\ O^{T} & 1 \end{array}\right]\left[\begin{array}{c} X_{w}\\ Y_{w}\\ Z_{w}\\ 1 \end{array}\right]\\ R=\left[\begin{array}{ccc} r_{1} & r_{2} & r_{3}\\ r_{4} & r_{5} & r_{6}\\ r_{7} & r_{8} & r_{9} \end{array}\right],T=\left[\begin{array}{c} T_{x}\\ T_{y}\\ T_{z} \end{array}\right],A=\left[\begin{array}{ccc} \alpha & \delta & u_{0}\\ 0 & \beta & v_{0}\\ 0 & 0 & 1 \end{array}\right] \end{array},$$

where T and R represent the translation vector and rotation matrix from the coordinate system to the CCS, respectively. α an β are the scale factors in u and v axes of the camera, respectively, and δ is the skew of the two image axes. ρ is a nonzero factor, and $\left(u_{0},v_{0}\right)$ is the principal point. 

The rotation matrix R and translation vector T, which translate to a 3D point $P_{c}(x_{c},y_{c},z_{c})$ in the CCS, encapsulate the camera orientation and position. The transformation relation of the CCS to the image coordinate system can be shown as 

(2) $$\rho \left[\begin{array}{c} u\\ \nu \\ 1 \end{array}\right]=\left[\begin{array}{ccc} \alpha & \delta & u_{0}\\ 0 & \beta & \nu_{0}\\ 0 & 0 & 1 \end{array}\right]\left[\begin{array}{c} X_{c}\\ Y_{c}\\ Z_{c} \end{array}\right],$$

Equation (2) shows the expression of a straight line in space, which connects the point in CCS with the point in the image plane. Practically, radial and tangential distortions of the lens are inevitable. In our practical engineering application, the tangential distortion of the lens has a minimal effect on the result. In this study, we only consider the radial distortion and we have the following equations:

(3) $$\{\begin{array}{c} \bar{x}=x(1+k_{1}r^{2}+k_{2}r^{4})\\ \bar{y}=y(1+k_{1}r^{2}+k_{2}r^{4}) \end{array},$$

where $r^{2}=x^{2}+y^{2}$, ${\left(x,y\right)}^{T}$ is the distorted image coordinate and ${\left(\bar{x},\bar{y}\right)}^{T}$ is the idealized one. $k_{1}$ and $k_{2}$ are the radial distortion coefficients of the lens. 

**Figure 7:**
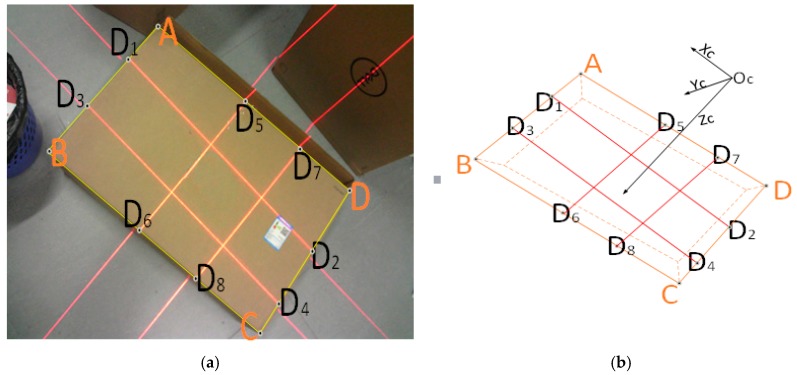
Key points in the image and camera coordinate system (CCS): (**a**) image coordinate system, and (**b**) CCS.

The laser light plane that is emitted from the visual sensor intersects with the box face and forms laser stripes in the image plane captured by the camera, as shown in Figure 7a. Assume that we have obtained (u,v) of the eight key points (D_1_–D_8_) on the laser line, as shown in Figure 7a. Section 3.4 presents the method of obtaining the eight key points in detail. Subsequently, we can obtain the spatial coordinates of key points (D_1_–D_8_) in the CCS, as shown in Figure 7b. Points A, B, C, and D are the four vertices of the measured box face.

Point $D_{1}$ in the image not only belongs to the intersection line with the surface to be digitized, but also to the laser light plane must fulfil the camera model equations. Once the perspective projection matrix of the camera and the equations of the planes containing the sheets of light relative to a global coordinate frame are obtained from the calibration, the triangulation for computing the 3D coordinates of object points simply involves finding the intersection of a ray from the camera and a plane from the projector. Thus, the equation of the laser plane in the CCS is as follows:

(4) $$a_{i}x_{c}+b_{i}y_{c}+c_{i}z_{c}+d_{i}=0,$$

where i is the laser stripe number and $a_{i}$, $b_{i}$, $c_{i}$, and $d_{i}$ are the coefficients. The number of equations of the planes and light stripes is equal. The laser plane contributes with the additional information that is necessary for completing the equation of the straight line of the camera model, such that their 3D coordinates can be extracted from their 2D image coordinates u, v.

A 3D point $P(x_{c},y_{c},z_{{}_{c}})$ at the intersection of the viewpoint from the camera and the laser stripe from the projector is triangulated while using the camera and projector parameters. On the basis of Equations (2) and (4), we derive the set of linear equations $\left[X_{c}/Y_{c},Y_{c}/Z_{c},1/Z_{c}\right]$, as follows:

(5) $$\left[\begin{array}{ccc} \alpha & \delta & 0\\ 0 & \beta & 0\\ a_{i} & b_{i} & d_{i} \end{array}\right]\left[\begin{array}{c} X_{c}/Z_{c}\\ Y_{c}/Z_{c}\\ 1/Z_{c} \end{array}\right]=\left[\begin{array}{c} u-u_{0}\\ v-v_{0}\\ -c_{i} \end{array}\right],$$

Therefore, $P(x_{c},y_{c},z_{c})$ in the CCS can be expressed as

(6) $$X_{c}=Z_{c}\frac{\left(u-u_{0})-\frac{\delta }{\beta }(v-v_{0}\right)}{\alpha },$$

(7) $$Y_{c}=Z_{c}\frac{\left(v-v_{0}\right)}{\beta },$$

(8) $$Z_{c}=\frac{d_{i}}{a_{i}}{\left(-\frac{c_{i}}{a_{i}}-\frac{\left(u-u_{0})-\frac{\delta }{\beta }(v-v_{0}\right)}{\alpha }-\frac{b_{i}}{a_{i}}\frac{\left(v-v_{0}\right)}{\beta }\right)}^{-1},$$

On the basis of the intersection of lines $D_{1}D_{3}$ and $D_{5}D_{7}$ in the CCS, the coordinate of intersection point A could be obtained as $A(X_{ca},Y_{ca},Z_{ca})$. Similarly, we can generate the 3D coordinates of B,C,D in the CCS: $B(X_{cb},Y_{cb},Z_{cb})$, $C(X_{cc},Y_{cc},Z_{cc})$, and $D(X_{cd},Y_{cd},Z_{cd})$. Thus, we derive the length and width of this box side.

(9) $$\begin{array}{l} width=1/2(\sqrt{{\left(X_{ca}-X_{cb}\right)}^{2}+{\left(Y_{ca}-Y_{cb}\right)}^{2}+{\left(Z_{ca}-Z_{cb}\right)}^{2}}+\\ \;\sqrt{{\left(X_{cd}-X_{cc}\right)}^{2}+{\left(Y_{cd}-Y_{cc}\right)}^{2}+{\left(Z_{cd}-Z_{cc}\right)}^{2}})\\ length=1/2(\sqrt{{\left(X_{ca}-X_{cd}\right)}^{2}+{\left(Y_{ca}-Y_{cd}\right)}^{2}+{\left(Z_{ca}-Z_{cd}\right)}^{2}}+\\ \;\sqrt{{\left(X_{cb}-X_{cc}\right)}^{2}+{\left(Y_{cb}-Y_{cc}\right)}^{2}+{\left(Z_{cb}-Z_{cc}\right)}^{2}}) \end{array},$$

Similarly, we capture the box’s image of the adjacent face to the first image. On the basis of Equation (9), we can measure the length and width of the second image: $width^{\prime }$ and $length^{\prime }$. Hence, the box height can be calculated.

(10) $$height=\{\begin{array}{l} width^{\prime }(min[(width^{\prime }-width),(width^{\prime }-length)]\\ \;<min[(length^{\prime }-width),(length^{\prime }-length)])\\ height^{\prime }(min[(width^{\prime }-width),(width^{\prime }-length)]\\ \;>min[(length^{\prime }-width),(length^{\prime }-length)]) \end{array},$$

Therefore, we can obtain the box volume.

(11) V=width*length*height.

However, a dimension of A*A*B of the measured box is a problem. At this time, if the two captured images that were calculated with the length of the box’s faces are A*B, then our algorithm will not work properly. At this point, we obtain the box length and width through the first image, but we cannot calculate the box height from the second image through Equation (10). As the values of A and B calculated by the second image satisfy Equation (10), we must manually select a suitable A or B as the box height in our system.

To date, a box volume measurement approach, which only requires two laser-modulated images of boxes, has been introduced. Section 3.3 designs a one-step calibration method for camera and laser projector. The coordinates of key points, which are automatically obtained by deep learning for laser-modulated image, are presented in Section 3.4.

### 3.3. Calibration Method for the Camera and 2 × 2 Laser Line Grid Projector

In this work, we present a one-step intrinsic and extrinsic calibration method for line-structured light projector that is based on circle calibration target. The coordinates of the key points are solved by increasing the equation of the laser plane. 

Zhang et al. [17] provided an excellent method for camera calibration. Line-structured light projector calibration involves determining the camera’s intrinsic and extrinsic parameters. Equation (1) represents a camera perspective projection model. The 3×3 rotation matrix R and 3×1 translation vector T are the external parameters of the camera. The laser plane (Equation (4)) in this coordinate system is obtained during line-structured light projector calibration. Here, we simultaneously generate the system parameters of the camera and the laser projector.

**Figure 8:**
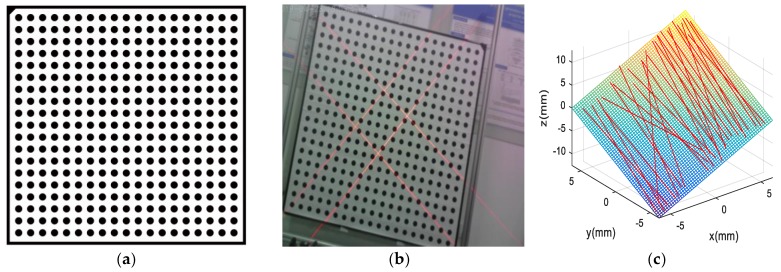
Calibration of the visual sensor: (**a**) circle calibration target; (**b**) calibration image; and, (**c**) laser plan fitting.

Figure 8a shows the circle target that is used in this paper. The visual sensor is placed at a distance from the target board similar to the nominal working distance. N images with different positions, which contain the laser line corresponding to the intersection of the laser plane with the calibration board, are captured (Figure 8b). We select the first local WCS as the absolute WCS from the N local WCSs previously established. The X and Y axes of each moving target are used as the local WCS to calculate the relative position between the CCS and local WCS $R_{i}$ and $T_{i}$. The laser plane (Equation (4)) is fitted in the absolute CCS (Figure 8c).

Therefore, the equation coefficients of the *i*th plane ($a_{i}$, $b_{i}$, $c_{i}$, and $d_{i}$) can be computed while using the least squares method. We obtain the line-structured light projector parameters on the basis of the circle calibration target by one step. Moreover, the proposed approach does not need to extract the standard points, but the inputs all coordinates of the laser stripes converted into the CCS. Therefore, the number of calibrated points is sufficient for the calibration of the laser plane. Subsequently, the equation of the laser plane is fitted to reduce the error. 

The calibration board is 1300 × 1200 × 5.0 mm, and N(N=28) images with different poses calibrate the system. The circle calibration target is printed with a high-quality printer and then placed on glass. Table 1 lists the detailed parameters of the camera and laser projector. Table 2 presents the calibration parameters.

### 3.4. Laser-Modulated Image Processing

#### 3.4.1. IHED Network for Extracting the Edge of the Laser-Modulated Image

**Figure 9:**
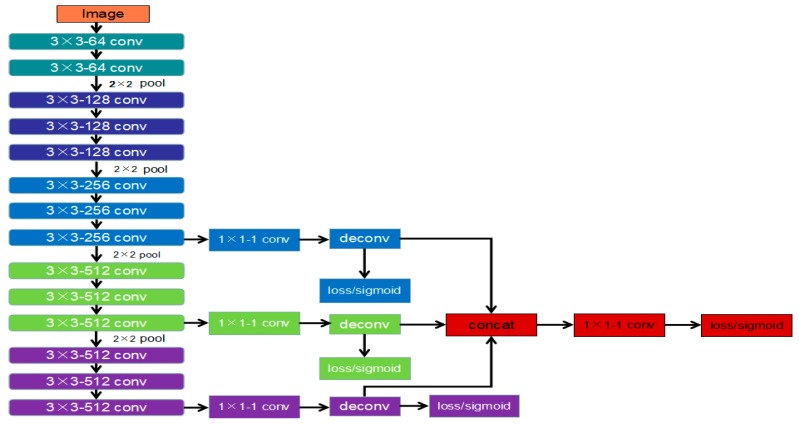
Architecture of the proposed improved holistically nested edge detection (IHED) network.

Variation in box materials, color, and appendages and the box texture influence laser-modulated images. The actual box edges and laser center lines are difficult to distinguish from lines in the laser-modulated images in complex scenarios. Although edge detection technology [33,34] can be used to find the box contour, these algorithms often perform particularly poorly in image processing in practical applications. Recently, FCNN has advanced in addressing the problem of detecting edge and object boundaries in natural images. Inspired by HED, we adopt a similar structure to the HED network and continuously inherit and learn the precise edge in the generated output process through the side output layer. We also design our network by modifying the VGG16 [35] network. Figure 9 displays the developed IHED network for edge detection. In comparison with HED, our modifications can be described, as follows:

1. To achieve the best edge detection effect, we build our own laser-modulated image dataset.
2. We cut the first two side output layers. Such an operation can remove considerable low-level edge information.
3. A cross-entropy loss/sigmoid layer is connected to the up-sampling layer in each stage without deep supervision.

**Figure 10:**
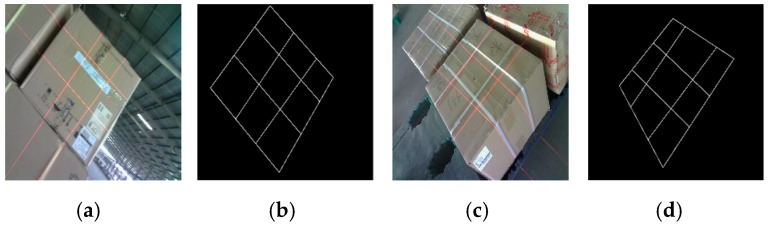
Two example images and ground-truth edge results for our dataset: (**a**,**c**) Input images; (**b**,**d**) ground-truth edges by human annotation of (**a**,**c**), respectively.

In total, 40,000 training images are obtained to determine the IHED network parameters and 1500 images are provided for testing. We manually mark the coordinate of the eight key points of the laser-modulated images and then draw straight lines to obtain the ground truth. Figure 10 shows two example images and the ground-truth edge results of the developed dataset.

In our IHED network, we consider the following objective function:

(12) $$L_{side}(W,w)=\sum_{m=1}^{M}\alpha_{m}l_{side}^{\left(m\right)}(W,w^{\left(m\right)}),$$

where $l_{side}$ denotes the image-level loss function for side outputs. W is the set representation of all standard network layer parameters. The parameters of side output are denoted as $w=(w^{\left(1\right)},\ldots ,w^{M})$, and the network has M side output layers.

In our network architecture, the loss function is computed over all the pixels in a training image $X=(x_{j},j=1,\ldots ,\left|X\right|)$ and edge map $Y=(y_{j},j=1,\ldots ,\left|Y\right|),y_{j}\in \{0,1\}$. In the training process, this cost function traverses every pixel of the input image and of the output probability graph. For each image, this function is defined as

(13) $$l_{side}^{\left(m\right)}(W,w^{\left(m\right)})=-\beta \sum_{j\in Y_{+}}\log\mathrm{Pr}(y_{j}=1|X;W,w^{\left(m\right)})-(1-\beta )\sum_{j\in Y_{\_}}\log\mathrm{Pr}(y_{j}=0|X;W,w^{\left(m\right)}),$$

where $\beta =\left|Y_{\_}\right|/\left|Y\right|$ and $1-\beta =\left|Y_{+}\right|/\left|Y\right|$. $Y_{+}$ and $Y_{\_}$ denote the edge and non-edge ground-truth label sets, respectively. At each side output layer, we obtain the edge probability map prediction ${\hat{Y}}_{side}^{\left(m\right)}=\sigma ({\hat{A}}_{side}^{\left(m\right)})$, where ${\hat{A}}_{side}^{\left(m\right)}\equiv \{\alpha^{\left(m\right)},j=1,\ldots ,|Y|\}$ are the activations of the side output of layer *m*.

Thus, the loss function for “weighted-fusion” layer is as follows:

(14) $$L_{fuse}(W,w)=Dis(Y,\sigma (\sum_{m=1}^{M}{\hat{A}}_{side}^{m})),$$

where σ(.) is the sigmoid function. Dis(.) is the distance between the fused predictions and ground-truth label map.

For all of these parameters, W, w is simultaneously optimized through standard backpropagation:

(15) $${\left(W,w\right)}^{*}=argmin(L_{fuse}(W,w)),$$

Hence, in the testing stage, given an image X, the final edge probability map can be defined as

(16) $${\hat{Y}}_{edge}=Average({\hat{Y}}_{fuse},{\hat{Y}}_{side}^{\left(3\right)},{\hat{Y}}_{side}^{\left(4\right)},{\hat{Y}}_{side}^{\left(5\right)}),$$

The network parameter settings are as follows: input image size (512 × 512), mini-batch size (9), learning rate (1 × 10^−3^), loss weight for each side output layer (1), weight decay (2 × 10^−4^), and number of training iterations (1 × 10^5^, learning rate is divided by 10 after 1000). This network design can not only realize high-precision and high-sensitivity edge detection, but also suppress internal texture edge.

A total of 1500 testing images are used to verify the effectiveness of our algorithm. This study uses the precision, recall, and F-measure to evaluate the edge detection performance of the laser-modulated image. The precision recall curve includes the recall rate and precision of the detection result. The precision reflects the pixel ratio of the used approach to extract the true structure edges (TP) and the total number of all detected edges. The recall rate reflects the TP and ground-truth edge. The F-measure is a comprehensive evaluation indicator with a fixed conversion relationship between recall and precision. The recall, precision, and F-measure are calculated, as follows:

(17) $$\mathrm{Precision}=\frac{\mathrm{TP}}{\mathrm{TP}+\mathrm{FP}},$$

(18) $$\mathrm{Recall}=\frac{\mathrm{TP}}{\mathrm{TP}+\mathrm{FN}},$$

(19) $$F-\mathrm{measure}=\frac{2*\mathrm{Precision}*\mathrm{Recall}}{\mathrm{Precision}+\mathrm{Recall}},$$

where FP is the wrong edge pixels that have been extracted and FN is the number of mis-extracted pixels.

**Figure 11:**
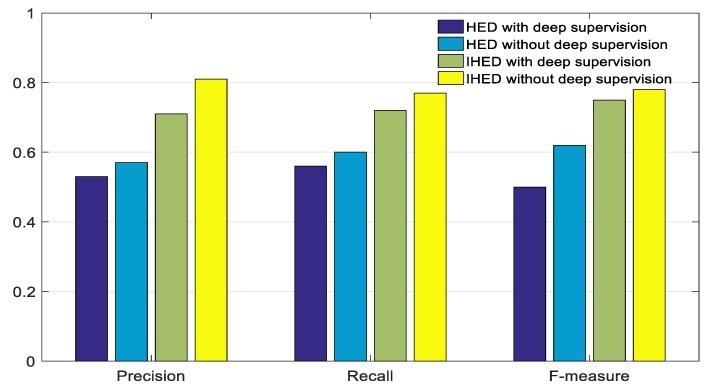
Performance comparison of the IHED and holistically nested edge detection (HED) networks with/without deep-supervision with respect to edge extraction.

The proposed IHED network without deep-supervision extraction of structure edges is compared with the HED algorithm to show its effectiveness. Figure 11 shows a performance comparison of these detection algorithms on our dataset with respect to the precision, recall, and F-measure of the extracted edges. The IHED without deep supervision has a better edge extraction performance than the other three network models.

**Figure 12:**
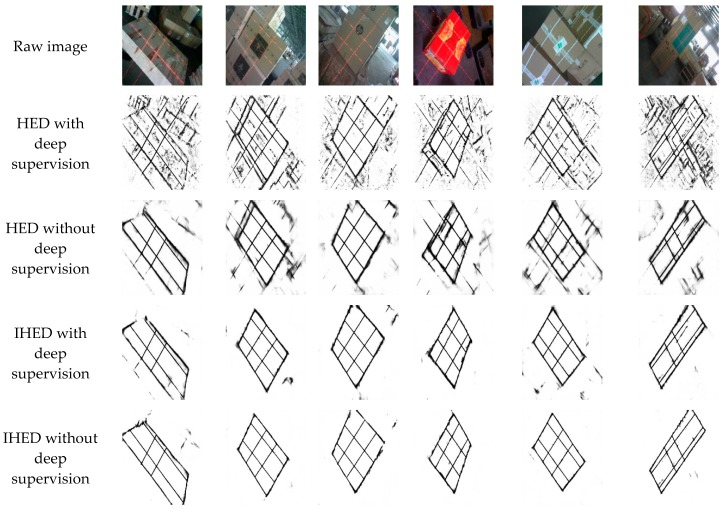
Edge detection results by the HED/IHED network with/without deep supervision.

Figure 12 shows several examples of edge detection on the dataset for the HED and IHED networks (network parameters are consistent). Rows 1, 2, 3, and 4 in Figure 12 display that IHED is more advantageous than HED in detecting the structural edge of the box. The HED network detects other non-box structure edges, which are avoided by the improved network (IHED). This result is consistent with the original intention of the edge detection of the design structure.

#### 3.4.2. Method for Extracting the 2D Coordinates of the Key Points of the Laser-Modulated Image

We must obtain the supporting lines for the edge probability maps to obtain the 2D coordinates of the box vertices. The edge probability map of the laser-modulated image has been obtained by our network (Section 3.4.1). By using the center coordinate of the image as the origin coordinate, we use the Hough line transform [36] to detect all the straight lines on the edge probability map. Equation (20) is used to represent them.

(20) ρ=xcos(θ)+ysin(θ),

Subsequently, we cluster the nearly collinear line segments by setting the suitable segmentation thresholds for ρ and θ (ρ∈[0pixel,15pixel] and $\theta \in [-1.8^{\circ },1.8^{\circ }]$ in this study). 

**Figure 13:**
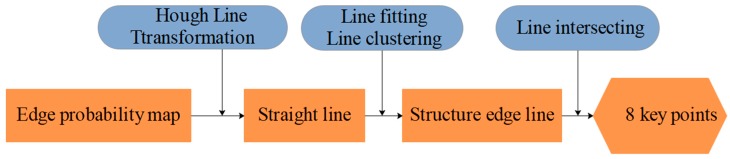
Edge probability map processing and key point extraction procedure.

**Figure 14:**
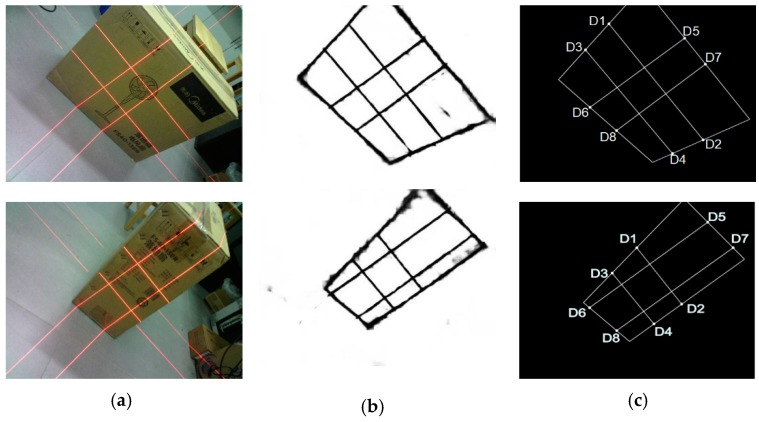
Verification of the accuracy of extracting pixel coordinates of eight key points; (**a**) original image (2592 × 1944); (**b**) edge probability map (512 × 512); and, (**c**) eight key points obtained by our approach.

We separately obtain the fitting line equation of the laser line and the edge of the measured box. Figure 13 shows the operation process. By finding the intersection points of these lines, the coordinates of eight key points on the 2D image can be deduced. Finally, we can easily locate the relationship of the eight key points (D1–D8) on the laser line through the geometric relationships between the box face’s edge and the laser line in the 2D image, as shown in Figure 14.

The original image resolution is 2592 × 1944 pixels and the size of the edge probability map output by the network is 512 × 512 pixels. Automatically extracting the eight key points in the collected box image with laser line has an important influence on the accuracy and automatic operation of the proposed system. We conduct pixel level coordinate error analysis between the raw image and edge probability image that were obtained through the IHED network. We convert the coordinates of the eight key points obtained to a camera resolution of 2592 × 1944. Here, we consider the maximum measuring range of the system to be 1800 mm. Thus, we can roughly estimate the actual physical distance of each pixel as $\frac{1800}{1944}$ mm. Assume that the maximum error allowed by the system is 5.0 mm. We can obtain the maximum pixel error that is allowed by the system as $5*\frac{1944}{1800}=5.40$ pixels. We analyze the pixel values of 1500 images in the test dataset.

(21) $$pixel\_error=\sum_{j=1}^{M}\sum_{i=1}^{N}\frac{1}{N}\left[\left(u_{i}-{u_{i}}^{\prime })+(v_{i}-{v_{i}}^{\prime }\right)\right],$$

where M is the number of test datasets. N is the number of key points on the image. In the experiment, M is 1500 and N is 8. (u,v) is the label pixel coordinate and $\left(u^{\prime },v^{\prime }\right)$ is the pixel coordinate that was obtained by our approach. The pixel coordinate error of key points is 1.96 < 5.40 pixels, which can meet our requirements.

## 4. Experiments

Figure 1c illustrates the system, wherein the device is connected with an android phone (HUAWEI honor Play) through a USB cable. The measurement environment parameters are as follows: temperature (−15~60 °C), measured distance from the visual sensor to the measured box (0.1–2.5 m), and measuring range of the box length, width, and height (10–1800 mm). The initial status calibration is performed before the experiment. Table 2 lists the calibration parameters of the visual sensor. 

Various experimental tests are conducted under varying operating conditions to test the robustness of the proposed system. Four experimental phases are performed to evaluate the system performances: (1) In Section 4.1, the measurement statistical analysis of boxes in complex scene is conducted. (2) In Section 4.2, the stability of the proposed system is verified. (3) In Section 4.3, the statistical analysis on real boxes is performed and the measurement uncertainty is evaluated by using the expression of uncertainty in measurement [37]. (4) In Section 4.4, the measurement error analysis of the optical quality of the boxes surface and the surface variation is performed. (5) In Section 4.5, the practical performance of the proposed system is evaluated in real-world tests.

### 4.1. Measurement Statistical Analysis of Boxes in Complex Scenarios

**Figure 15:**
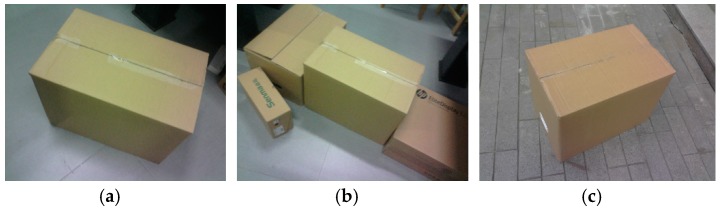
Measured box in different scenarios; (**a**–**c**) are three boxes in different scenarios.

The experiment tests the accuracy of the system’s measurements in complex and outdoor environments. Figure 15a shows a single box captured indoors, with a dimension of 490.7 mm × 560.5 mm × 651.0 mm. Figure 15b presents the box measurement in a complex indoor environment, with multiple interfering boxes that are near the measured box. Figure 15c exhibits the image captured outdoors, in which the laser line is dim in the image due to the influence of strong illumination. 

**Figure 16:**
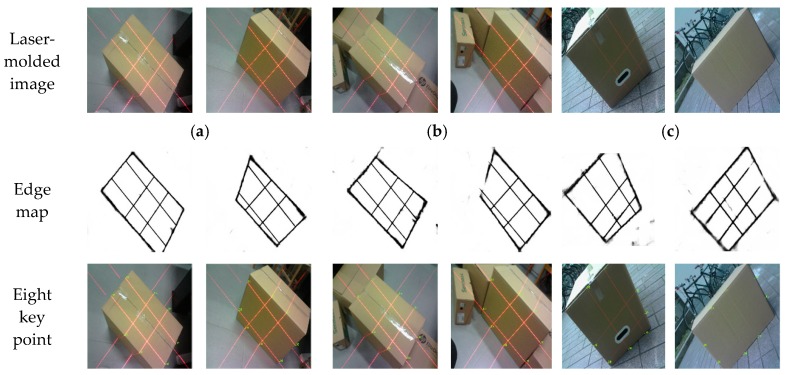
Image processing and key point extraction by our algorithm; (**a**–**c**) are the measured images captured by our device.

Figure 16 shows the measurement results of the box that was acquired in Figure 15. The edge probability map is obtained after processing the IHED network, and coordinates of the eight key points are determined. Even if the box images (Figure 16c) are collected outdoors, the edge probability map can be efficiently processed by our system.

**Figure 17:**
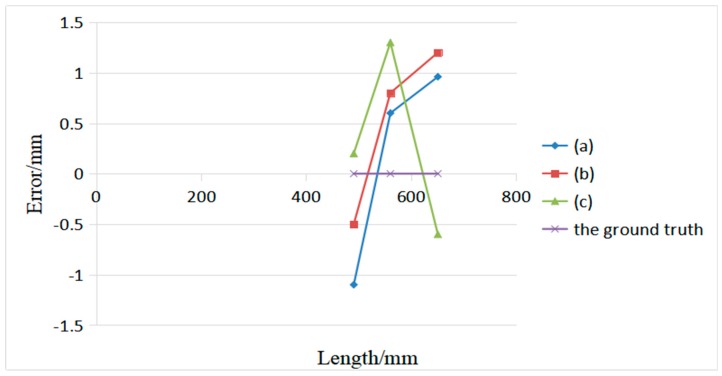
Measurement results of the box system in different scenarios.

The final estimated values are recorded as the average of three experimental sessions on the box. Figure 17 shows the measurement results and actual dimensions of the measured box under different scenarios. The maximum average absolute error is 1.3 mm. Hence, our volume measurement system can accurately measure the length of each side of the box in a complex environment, which can meet the actual measurement requirements.

### 4.2. Pose Stability Testing

**Figure 18:**
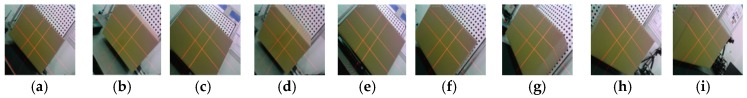
Images of nine different poses; (**a**) vertical shooting; (**b**) tilt 30° to the left; (**c**) tilt 60° to the left; (**d**) tilt 30° to the right; (**e**) tilt 60° to the right; (**f**) tilt 30° upward; (**g**) tilt 60° upward; (**h**) tilt 30° downward; and, (**i**) tilt 60° downward.

**Figure 19:**
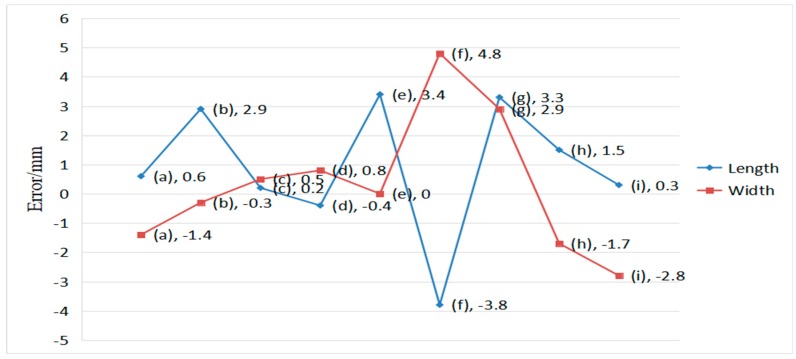
Errors between the standard box and the measured result.

This experiment aims to verify the stability of the measured box from different viewpoints. As shown in Figure 18, the box is measured from different angles with nine poses to simulate the pose difference in actual measurement. In this experiment, the volume measurement system is used to obtain the box length and width under different poses. Only one face of the standard box (800 mm × 600 mm) is measured in this experiment to facilitate measurement and comparison. Estimated values are reported as the average of 30 experimental sessions on the same surface (800 mm × 600 mm) in Table 3. The relative errors are generally relatively small. The deviation between the estimated and actual values is within ±5.0 mm at each pose. The pose of the visual device appears to have minimal effect on the measurement accuracy of the proposed system on the basis of the mean error analysis in Table 3. The proposed system can effectively handle the measured certainty, regardless of which view the images are captured with strict measurement rules. The values of standard deviations are 1.7521 and 1.7175 mm respectively, which indicates that the box measurement system has reliable repeated measurement accuracy. Figure 19 shows that the length errors of the box dimensions are within 5.0 mm. The results show that the system stability is remarkable.

### 4.3. Error Analysis on Real Box and the Evaluation of Uncertainty in the Measurement Result of Box Volume

This volume measurement system can calculate the dimension parameters of the box simply via laser triangulation and deep learning technology; thus, the entire system maintains the advantages of simple configuration and low cost. However, this method includes three main factors that affect the measurement accuracy of the box length: the measurement error of the visual sensor and the position error of the box (the distance and pose between the measured box and visual sensor). We conduct statistical experiments to evaluate the effectiveness of the method.

**Figure 20:**
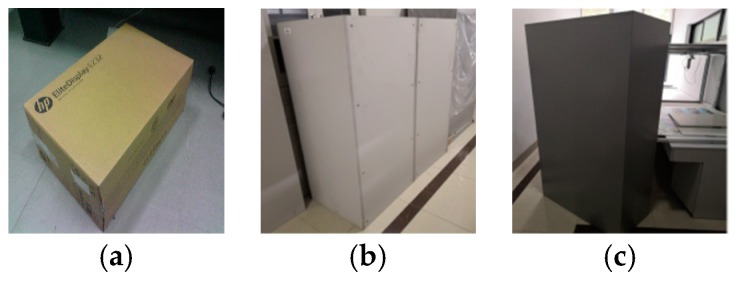
Examples of real standard boxes used for testing; (**a**–**c**) are the boxes of #1, #2, #3 respectively.

As shown in Figure 20, the three standard boxes (#1, #2, and #3) are selected in the experiment. Their length, width, and height are 330.4 × 110.3 × 440.6, 690.7 × 570.5 × 1500.0, and 900.0 × 400.0 × 1800.0, respectively. We use our system to collect 15 measurements for each of the three standard boxes (Table 4). We utilize these data to calculate the mean and standard deviation of each box’s side length.

The data of the measurement results in Table 4 are statistically analyzed to evaluate the measurement accuracy scientifically, and the uncertainty of class A ($\mu_{A}$) is calculated as

(22) $$\mu_{A}=\sqrt{\frac{\sum_{i=1}^{n}{\left(x_{i}-\bar{x}\right)}^{2}}{n-1}},$$

where $x_{i}$ is the estimated length and $\bar{x}$ is the mean value of the measured data. n is the number of measurements, which is 15 in this study.

**Figure 21:**
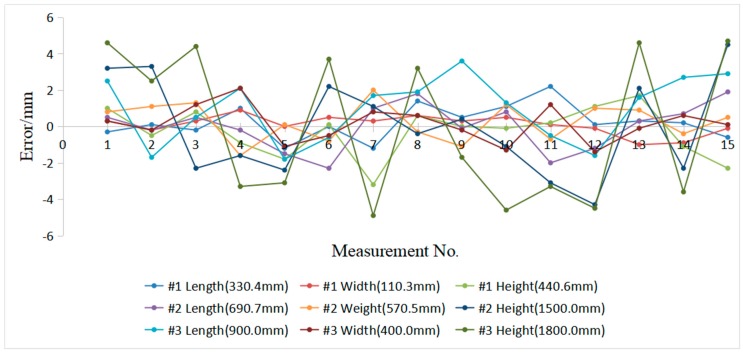
Errors between the standard box and the measured result.

**Figure 22:**
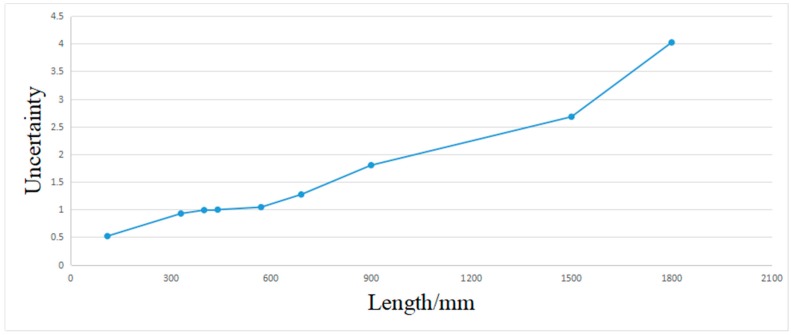
The relationship between the measurement uncertainty and the box length.

Table 4 shows the measurements result, with a minimum uncertainty of ±0.52 mm and maximum uncertainty of ±4.0 mm. The measurement uncertainty in the estimated length increases with the length. The measurement uncertainty is in accordance with the experiment that is described in Table 4. Figure 21 shows that the length errors of the box dimensions are within ±5.0 mm. The results show that the system has good accuracy. Figure 22 shows the measurement uncertainty of the measuring device, which is consistent with the experimental results.

### 4.4. Measurement Error Analysis of the Optical Quality of the Boxes Surface and the Surface Variation

**Figure 23:**
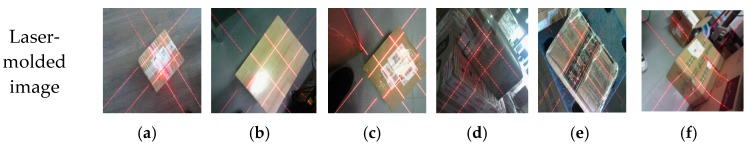

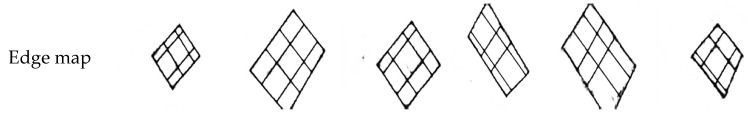
Image processing by our algorithm; (**a**–**c**) exhibit the images captured at different optical quality; (**d**–**f**) exhibit the images captured the boxes with surface variation.

The experiment tests the effect of the system’s measurements on the optical quality of the boxes surface and the surface variation. Figure 23 shows the boxes, and only one face of the box is measured in this experiment: (a) 350.2 mm × 260.5 mm, (b) 376.5mm × 276.4 mm, (c) 340.4 mm × 420.6 mm, (d) 560.0 mm × 380.0 mm, (e) 480.6 mm × 365.7 mm, and (f) 300.6 mm × 250.0 mm. Figure 23a–c exhibit the images captured at different optical quality. Figure 23d–f test boxes with surface variation. The second row in Figure 23 shows the image processing results of the boxes faces. 

**Figure 24:**
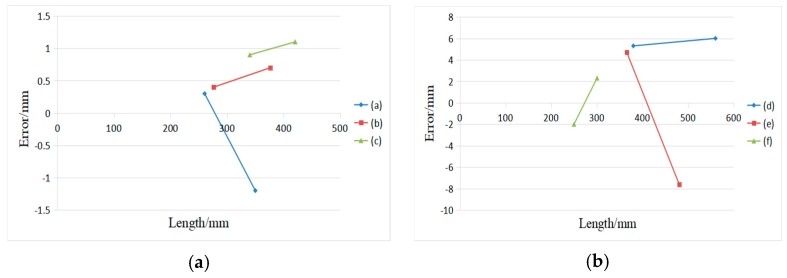
Measurement results; (**a**) Errors between the actual length and the measured result affected by optical quality of the box surface; and, (**b**) Errors between the actual length and the measured result tested on boxes with surface variation.

Figure 24a shows the measurement results of the optical quality of the boxes surface, with a minimum measurement error of 0.2 mm and maximum error of 1.3 mm. Figure 24b shows the measurements result of the surface variation, with a minimum measurement error of 2.0 mm and maximum error of 7.6 mm. The results show that the system suffered little from the optical quality of the surface, but it has big uncertainty when measuring the surface variation of the box. 

### 4.5. Online Measurement Testing

**Figure 25:**
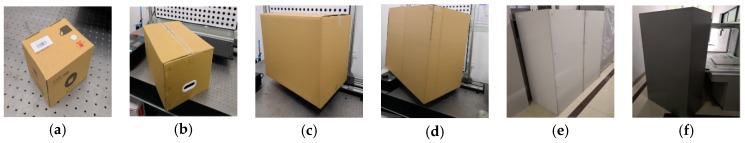
Six standard boxes with different dimension parameters: (**a**) 143.4 × 120.5 × 100; (**b**) 550.6 × 350.5 × 300.0; (**c**) 800.0 × 600.0 × 500.0; (**d**) 1200.0 × 900.0 × 700.0; (**e**) 1500.0 × 690.7 × 570.5; and, (**f**) 1800.0 × 900.0 × 400.0.

Six standard boxes with different sizes and volumes are selected for measurement to evaluate the measurement accuracy scientifically, as shown in Figure 25. Table 5 displays the corresponding experimental results. The final measurement of the box length is highlighted in bold. We estimate of the relative measurement error of the volume $\varepsilon =\left|v_{e}-v_{a}\right|/v_{a}$, where $v_{e}$ is the estimated volume and $v_{a}$ is the value of actual volume. The results in Table 5 indicate that the error of the measurement system increases with the side length of the measured box, but the error range of the measured and actual values of the single side length of each standard box is within ±5.0 mm. The maximum relative measurement error of the volume (ε) of the measured box is 2.27% and the mean relative error is 0.83%, which indicates good precision.

## 5. Conclusions

This research presents a line-structured light-based 3D measuring sensor and deep-learning-based box volume measuring method. Our box volume measurement method only requires two laser-modulated images. We propose a novel end-to-end edge detection architecture based on an IHED network to extract the structure straight edge lines in laser-modulated images. By cutting the first two side output layers and training without deep supervision of HED, our network can learn robust straight line features from laser-modulated images. Moreover, we present a one-step calibration method to calibrate our portable measuring sensor automatically. Experimental results show that the measuring range of our proposed system is 100–1800 mm with errors less than ±5.0 mm. Our system is suitable for portable automatic box volume measurement, and it is useful for warehouses and distribution and logistics companies. Our future work will focus on small portable measuring devices.

## Figures and Tables

**Table 1:** Detailed parameters of the experimental equipment.

Device	Picture of Real Products	Parameters	Number
Digital color camera	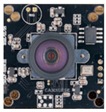	CCD: S-YUE, 1/1.8″ Resolution: 2592 (H) × 1944 (V) Pixel size: 4.4 μm × 4.4 μm Frame rate: 15 fps Focal length: 3.6 mm Signal-to-noise ratio: 50 db Field of view: 71.9° × 60.4° Size: 32 × 32 × 22 mm Operation temperature: −20 °C–60 °C Shooting distance: 50 mm~inf.	1 pcs
Laser line projector	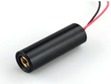	Power: 10 mW (adjustable) Focal length: adjustable Wavelength: 635 nm Size: *ϕ*5 × 20 mm Fan angle: 60° temperature: −20 °C–60 °C	4 pcs

**Table 2:** Calibration parameters of the structured optical system.

Title	Value
Camera intrinsic	$A=\left[\begin{array}{ccc} 2458.9172 & 0 & 1239.5188\\ 0 & 2453.8100 & 1032.5590\\ 0 & 0 & 1 \end{array}\right]$
Distortion coefficients	k1 = −0.03415937, k2 = 0.321070446
Pixel error	[0.0654, 0.0845]
Laser projector parameters	0.00792910x + (−0.00817394)y + 0.00018065z = 1
	0.01078476x + (−0.01121147)y + 0.00223845z = 1
	0.01037354x + 0.00959161y + 0.00170705z = 1
	0.00799885x + 0.00730513y + (−0.00038506)z = 1

**Table 3:** Error analysis of the measurement results of nine different poses for 30 times.

Pose	Actual Length/mm	Average Estimated Length/mm	Error (Length)/mm	Actual Width/mm	Average Estimated Width/mm	Error (Width)/mm
(a)	800	800.6	+0.6	600	598.6	−1.4
(b)		802.9	+2.9		599.7	−0.3
(c)		800.2	+0.2		600.5	+0.5
(d)		799.6	−0.4		600.8	+0.8
(e)		803.4	+3.4		600.0	+0.0
(f)		796.2	−3.8		604.8	+4.8
(g)		803.3	+3.3		602.9	+2.9
(h)		798.5	+1.5		598.3	−1.7
(i)		799.7	+0.3		597.2	−2.8
standard deviations		1.7521			1.7175	

**Table 4:** Measurement results of the system of three standard boxes (mm).

No.	Length (#1)	Width (#1)	Height (#1)	Length (#2)	Width (#2)	Height (#2)	Length (#3)	Width (#3)	Height (#3)
1	330.1	110.6	441.6	691.2	571.3	1503.2	902.5	400.3	1804.6
2	330.5	110.1	440.1	690.5	571.6	1503.3	898.3	399.8	1802.5
3	330.2	110.6	441.4	691.2	571.8	1497.7	900.5	401.2	1804.4
4	331.4	111.2	439.7	690.5	568.9	1498.4	902.1	402.1	1796.7
5	329.2	110.3	438.8	689.2	570.6	1497.6	898.2	398.9	1796.9
6	330.4	110.8	440.7	688.4	569.7	1502.2	899.4	399.5	1803.7
7	329.2	110.6	437.4	691.7	572.5	1501.1	901.7	400.8	1795.1
8	331.8	110.9	441.2	692.5	570.2	1499.6	901.9	400.6	1803.2
9	330.9	110.6	440.6	690.6	569.4	1500.4	903.6	399.8	1798.3
10	331.5	110.8	440.5	691.5	571.7	1498.9	901.3	398.7	1795.4
11	332.6	110.4	440.8	688.7	569.8	1496.9	899.5	401.2	1796.7
12	330.5	110.2	441.7	689.5	571.5	1495.7	898.4	398.6	1795.5
13	330.7	109.3	442.3	691	571.4	1502.1	901.6	399.9	1804.6
14	330.6	109.4	439.5	691.4	570.1	1497.7	902.7	400.6	1796.4
15	329.8	110.2	438.3	692.6	571.0	1504.5	902.9	400.1	1804.7
Mean	330.6	110.4	440.3	690.7	570.7	1500.0	901.0	400.1	1799.9
Standard deviation	0.8952	0.5007	0.9630	1.2285	1.0066	2.5868	1.7401	0.9550	3.8859
Uncertainty	0.9266	0.5182	0.9968	1.2717	1.042	2.6776	1.8012	0.9885	4.0223

**Table 5:** Volume measurement results in real applications.

	Box	Edge Probability Map	Actual Length/mm	Estimated Length/mm	Error Length/mm	Actual Volume/m^3^	Estimated Volume/m^3^	Relative Error/%
(a)	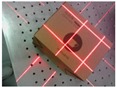	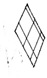	143.4	142.2	−1.2	0.0017	0.00169	2.27
			120.5	119.6	−0.9			
	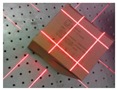	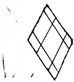	120.5	119.2	−1.3			
			100.0	99.3	−0.7			
(b)	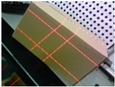	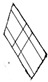	550.6	552.3	1.7	0.0579	0.05779	0.18
			300.0	299.3	−0.7			
	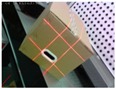	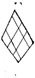	300.0	301.2	1.2			
			350.5	349.6	−0.9			
(c)	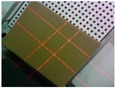	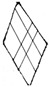	800.0	801.6	1.6	0.2400	0.24171	0.72
			500.0	503.5	3.5			
	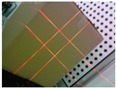	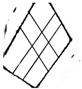	500.0	504.6	4.6			
			600.0	598.9	−1.1			
(d)	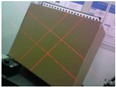	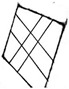	1200.0	1204.6	4.6	0.7560	0.76096	0.66
			900.0	898.6	−1.4			
	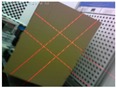	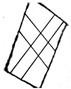	900.0	903.4	3.4			
			700.0	703.0	3.0			
(e)	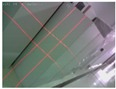	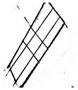	570.5	569.0	−1.5	0.5911	0.59221	0.19
			1500.0	1502.3	2.3			
	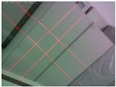	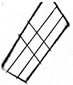	1500.0	1497.6	−2.4			
			690.7	692.8	2.1			
(f)	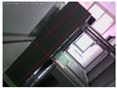	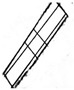	400.0	397.6	−2.4	0.6480	0.64179	0.96
			1800.0	1805.0	5.0			
	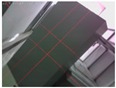	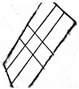	1800.0	1795.5	−4.5			
			900.0	899.0	−1.0			

## References

[1] Park H.M., Van Messemac A., De Neveac W. Box-Scan: An efficient and effective algorithm for box dimension measurement in conveyor systems using a single RGB-D camera. Proceedings of the 7th IIAE International Conference on Industrial Application Engineering.

[2] Chan T., Lichti D., Jahraus A., Esfandiari H., Lahamy H., Steward J., Glanzer M. (2018). An Egg Volume Measurement System Based on the Microsoft Kinect. Sensors.

[3] Andújar D., Dorado J., Fernández-Quintanilla C., Ribeiro A. (2016). An approach to the use of depth cameras for weed volume estimation. Sensors.

[4] Leo M., Natale A., Del-Coco M., Carcagnì P., Distante C. (2017). Robust estimation of object dimensions and external defect detection with a low-cost sensor. J. Nondestruct. Eval..

[5] Ferreira B.Q., Griné M., Gameiro D., Costeira J.P., Santos B.S. (2014). VOLUMNECT: Measuring volumes with Kinect. Three-Dimensional Image Processing, Measurement (3DIPM), and Applications 2014.

[6] Wang W., Liu P., Ying R., Wang J., Qian J., Jia J., Gao J. (2019). A High-Computational Efficiency Human Detection and Flow Estimation Method Based on TOF Measurements. Sensors.

[7] Wang Z., Walsh K., Verma B. (2017). On-tree mango fruit size estimation using RGB-D images. Sensors.

[8] Mustafah Y.M., Noor R., Hasbi H., Azma A.W. Stereo vision images processing for real-time object distance and size measurements. Proceedings of the 2012 International Conference on Computer and Communication Engineering (ICCCE).

[9] Ge L., Yang Z., Sun Z., Zhang G., Zhang M., Zhang K., Zhang C., Tan Y., Li W. (2019). A method for broccoli seedling recognition in natural environment based on binocular stereo vision and gaussian mixture model. Sensors.

[10] Makhsous S., Mohammad H.M., Schenk J.M., Mamishev A.V., Kristal A.R. (2019). A Novel Mobile Structured Light System in Food 3D Reconstruction and Volume Estimation. Sensors.

[11] Shang J., Duong M., Pepin E., Zhang X., Sandara-Rajan K., Mamishev A., Kristal A. A mobile structured light system for food volume estimation. Proceedings of the 2011 IEEE International Conference on Computer Vision Workshops (ICCV Workshops).

[12] Sarbolandi H., Lefloch D., Kolb A. (2015). Kinect range sensing: Structured-light versus Time-of-Flight Kinect. Comput. Vis. Image Underst..

[13] Liberadzki P., Adamczyk M., Witkowski M., Sitnik R. (2018). Structured-Light-Based System for Shape Measurement of the Human Body in Motion. Sensors.

[14] Fernandes L.A., Oliveira M.M., da Silva R., Crespo G.J. (2006). A fast and accurate approach for computing the dimensions of boxes from single perspective images. J. Braz. Comput. Soc..

[15] Wang X., Xie Z., Wang K., Zhou L. (2018). Research on a Handheld 3D Laser Scanning System for Measuring Large-Sized Objects. Sensors.

[16] Pan X., Liu Z., Zhang G. (2018). Reliable and Accurate Wheel Size Measurement under Highly Reflective Conditions. Sensors.

[17] Zhang Z.Y. (2000). A flexible new technique for camera calibration. IEEE Trans. Pattern Anal. Mach. Intell..

[18] Santolaria J., Guillomía D., Cajal C., Albajez J.A., Aguilar J.J. (2009). Modelling and calibration technique of laser triangulation sensors for integration in robot arms and articulated arm coordinate measuring machines. Sensors.

[19] Li Y.F., Chen S.Y. (2003). Automatic recalibration of an active structured light vision system. IEEE Trans. Robot. Autom..

[20] An Y., Bell T., Li B., Xu J., Zhang S. (2016). Method for large-range structured light system calibration. Appl. Opt..

[21] Zhang G., Liu Z., Sun J., Wei Z. (2010). Novel calibration method for a multi-sensor visual measurement system based on structured light. Opt. Eng..

[22] Bazargani H., Laganière R. (2015). Camera calibration and pose estimation from planes. IEEE Instrum. Meas. Mag..

[23] Rodríguez J.A.M., Mejía Alanís F.C. (2016). Binocular self-calibration performed via adaptive genetic algorithm based on laser line imaging. J. Mod. Opt..

[24] Muñoz-Rodriguez J.A. (2010). Mobile calibration based on laser metrology and approximation networks. Sensors.

[25] Muñoz-Rodriguez J.A. (2018). Microscope self-calibration based on micro laser line imaging and soft computing algorithms. Opt. Lasers Eng..

[26] Long J., Shelhamer E., Darrell T. (2014). Fully convolutional networks for semantic segmentation. IEEE Trans. Pattern Anal. Mach. Intell..

[27] Shen W., Wang X., Wang Y., Bai X., Zhang Z. Deepcontour: A deep convolutional feature learned by positive-sharing loss for contour detection. Proceedings of the IEEE Conference on Computer Vision and Pattern Recognition.

[28] Hallman S., Fowlkes C.C. Oriented edge forests for boundary detection. Proceedings of the IEEE Conference on Computer Vision and Pattern Recognition.

[29] Liu Y., Cheng M.M., Hu X., Wang K., Bai X. Richer convolutional features for edge detection. Proceedings of the IEEE Conference on Computer Vision and Pattern Recognition.

[30] Song W., Zhong B., Sun X. (2019). Building Corner Detection in Aerial Images with Fully Convolutional Networks. Sensors.

[31] Xie S., Tu Z. Holistically-nested edge detection. Proceedings of the IEEE International Conference on Computer Vision.

[32] Zhou P., Xu K., Wang D. (2018). Rail profile measurement based on line-structured light vision. IEEE Access.

[33] Canny J. (1986). A computational approach to edge detection. IEEE Trans. Pattern Anal. Mach. Intell..

[34] Martin D., Fowlkes C., Malik J. (2004). Learning to detect natural image boundaries using local brightness, color, and texture cues. IEEE Trans. PAMI.

[35] Simonyan K., Zisserman A. (2014). Very deep convolutional networks for large-scale image recognition. arXiv.

[36] Duda R.O., Hart P.E. (1971). Use of the Hough Transformation to Detect Lines and Curves in Pictures (No. SRI-TN-36).

[37] Kirkup L., Frenkel R.B. (2006). An Introduction to Uncertainty in Measurement: Using the GUM (Guide to the Expression of Uncertainty in Measurement).

